# Rapidly detecting fennel origin of the near-infrared spectroscopy based on extreme learning machine

**DOI:** 10.1038/s41598-022-17810-y

**Published:** 2022-08-10

**Authors:** Enguang Zuo, Lei Sun, Junyi Yan, Cheng Chen, Chen Chen, Xiaoyi Lv

**Affiliations:** 1 College of Information Science and Engineering, Xinjiang University, Urumqi, 830046 China; 2 College of Software, Xinjiang University, Urumqi, 830046 China; 3 Key Laboratory of signal detection and processing, Xinjiang University, Urumqi, 830046 China; 4 Xinjiang Uygur Autonomous Region Product Quality Supervision and Inspection Research Institute, Urumqi, 830011 China

**Keywords:** Health care, Risk factors

## Abstract

Fennel contains many antioxidant and antibacterial substances, and it has very important applications in food flavoring and other fields. The kinds and contents of chemical substances in fennel vary from region to region, which can affect the taste and efficacy of the fennel and its derivatives. Therefore, it is of great significance to accurately classify the origin of the fennel. Recently, origin detection methods based on deep networks have shown promising results. However, the existing methods spend a relatively large time cost, a drawback that is fatal for large amounts of data in practical application scenarios. To overcome this limitation, we explore an origin detection method that guarantees faster detection with classification accuracy. This research is the first to use the machine learning algorithm combined with the Fourier transform-near infrared (FT-NIR) spectroscopy to realize the classification and identification of the origin of the fennel. In this experiment, we used Rubberband baseline correction on the FT-NIR spectral data of fennel (Yumen, Gansu and Turpan, Xinjiang), using principal component analysis (PCA) for data dimensionality reduction, and selecting extreme learning machine (ELM), Convolutional Neural Network (CNN), recurrent neural network (RNN), Transformer, generative adversarial networks (GAN) and back propagation neural network (BPNN) classification model of the company realizes the classification of the sample origin. The experimental results show that the classification accuracy of ELM, RNN, Transformer, GAN and BPNN models are above 96%, and the ELM model using the hardlim as the activation function has the best classification effect, with an average accuracy of 100% and a fast classification speed. The average time of 30 experiments is 0.05 s. This research shows the potential of the machine learning algorithm combined with the FT-NIR spectra in the field of food production area classification, and provides an effective means for realizing rapid detection of the food production area, so as to merchants from selling shoddy products as good ones and seeking illegal profits.

## Introduction

As one of the popular spices that are widely used as condiments in daily life^1^, the fennel is widely planted all over the world^2^. In addition, the fennel can also be used as a raw material for the production of wine, creams, perfumes, biochemical materials, etc.^3–5^, and has some medicinal value. It can be used to prepare many drugs^6^, such as in the breeding industry and other fields. The antibacterial properties of fennel can replace antibiotics in feed additives and medicines in the poultry industry, and can effectively prevent the abuse of antibiotics^7^. On the other hand, the fennel and its derivatives have many beneficial medical properties, which can be used to treat digestive diseases such as epilepsy, anorexia and abdominal distension^8^, lower blood pressure, antibacterial and anti-inflammatory, prevent cancer, and treat diabetes^6,9,10^.

In recent years, a large number of researchers have focused on the chemical composition of the fennel because of its versatility, and found that the efficacy of the fennel and its products is related to the type and amount of the chemicals contained, such as antioxidant phenols and antibacterial terpenes. In addition, studies have shown that the relative concentration of compounds contained in fennel largely depends on its geographical origin^11^, because the types and content of biochemical components of fennel are origin dependent on geographical location^12^. Due to the influence of the external environment such as the climate, temperature, soil conditions, and precipitation of the production area, and the combined effect of internal and external factors such as genotypes^13,14^, the kinds and contents of substances and yields of fennel grown in different regions are significantly different^12,15–17^. The main biologically active ingredients contained in the fennel, such as antibacterial microorganisms, phenols and fatty acids, are different in content^12^, resulting in different quality, nutritional content and prices^16^. Yingdong College of Biological Engineering, Shaoguan University, Guangdong Province, China compared the nutrient content of 7 fennel varieties from Yumen, Gansu and Yili, Xinjiang, and comprehensive evaluation showed that the nutritional value of fennel varieties from Yumen, Gansu was relatively high. In order to prevent some merchants from using shoddy products as good ones and causing obstacles to market supervision, it is of great significance to accurately identify the origin of the fennel. However, the existing studies are mainly focused on the analysis of the differences in the kinds and contents of chemical substances in fennel from different regions^12,17^, and there are still gaps in the classification and identification of the origin of the fennel.

Different from the traditional food production area classification methods, such as molecular biotechnology, multi-element and multi-isotope analysis, which have complicated steps and high detection requirements^18,19^, the FT-NIR spectroscopy has the advantages of simple sample preparation and low detection cost^20^ In recent years, the FT-NIR spectroscopy combined with machine learning algorithms have been widely used in the field of food production area classification. Many research teams have completed the origin classification of tea, pistachio fruit, olive oil, multiflorum, cocoa bean, wheat, honey, radix glycyrrhizae and other foods by the FT-NIR combined with the machine learning algorithm^21–29^. As a nonlinear neural network, the BPNN (back propagation neural network) can solve complex problems more accurately than linear neural networks^28,30^. Xiu Ying Liang et al. used the BPNN combined with the FT-NIR spectroscopy to classify honey from different flower lines, the accuracy could reach 100% in a specific spectral segment^27^. Yan Tian ying et al. used the convolutional neural network (CNN) and recurrent neural network (RNN) models combined with the NIR spectroscopy to identify the geographical origins of Radix Glycyrrhizae from Gansu, Inner Mongolia, Ningxia, and Xinjiang, respectively, with the classification accuracy could reach 93%^29^. Yang et al. proved that when generative adversarial network (GAN) has a small amount of training data, the input data does not need feature selection, but also the model obtained by competitive learning is better than other classification algorithms, which provides a new method for the existing infrared spectrum research^28^. However, the time cost of GAN in the classification process is relatively high. For practical application scenarios, the detection data in the food production area classification is usually massive, which puts a higher demand on the detection time.

Compared with the traditional neural networks, ELM (extreme learning machine) has several remarkable characteristics: easy to use, faster-learning speed and higher generalization performance^31^. In short, the ELM-based model can be faster than the traditional learning algorithm on the premise of ensuring accuracy, so it is more suitable for the rapid detection of food production. Felix YH Kutsanedzie et al. used the ELM combined with the NIR spectroscopy to complete the classification of three grades of cocoa beans, with an accuracy of 94%^32^; Wenbin Zheng et al. found that the performance of the ELM was much better than that of the KNN, LS-SVM and BPNN in the application of the NIR spectroscopy for food classification, which indicates that the ELM may be a promising real-time food classification method^33^. Therefore, in this study, we aim to realize the rapid identification of fennel from different origins by the FT-NIR spectroscopy combined with the ELM and deep learning models, so as to provide an intelligent supervision method for the phenomenon of different the good and bad products in the fennel market caused by some merchants selling shoddy products.

## Materials and method

### Sample preparation and plant statement

The fennel seeds used in this experiment were harvested in 2020 and we purchased them in batches from local spice companies in Turpan, Xinjiang and Yumen, Gansu in different seasons to ensure that the experimental samples were from the origin and that the effects of different climatic conditions on the samples were taken into account. 200 samples were collected from Lianyungang Kaihao Tong Trading Co. in Xinjiang, and 116 samples were collected from Gansu Yumen Xiaosannong Taobao online store. Since the fennel seeds contain a variety of volatile components, such as essential oils, heating and drying should be avoided when storing the samples^34^. We stored the prepared samples in a dry and airtight atmosphere at room temperature for one week, then put them into the pulverizer. The sample powder was passed through a 200-mesh sieve and subsequently stored in a sealed self-sealing bag. It should be noted that we comply with relevant institutional, national, and international guidelines and legislation of this paper collected experiment sample.

### Spectral data collection and preprocessing

All samples in this experiment were measured indoors at a room temperature of 22$^{\circ }$C. Before each measurement, OPUS 65 software was used to measure the atmospheric background data under the Windows XP system environment, and then the background data of each sample was measured. Vertex 70 FT-NIR spectrometer (Bruker) was used in the experiment. $\mathrm {CO}_{2}$ compensation was selected as the atmospheric compensation parameter. The scanning parameters were as follows: the scanning range was 4000–11000 $\mathrm {cm}^{-1}$, the resolution was 8 $\mathrm {cm}^{-1}$, and the scanning was repeated 32 times. The spectrum data of 116 cases of fennel seed samples in Yumen, Gansu, and 200 cases of fennel seed samples in Turpan, Xinjiang were obtained. A total of 316 cases of FT-NIR spectrum data were obtained.

The original FT-NIR spectrum collected by the instrument contains many interference factors, which will affect the classification effect of the model^35,36^. Spectral preprocessing is to reduce or eliminate the influence of interference factors on the spectrum, thus improving the accuracy and reliability of the classification model. Baseline correction is one of the most commonly used methods in preprocessing. The experiment uses the Rubberband baseline correction method, and the baseline point value is 64. In addition, the environmental factors during spectral data collection and preprocessing were strictly consistent to ensure that environmental differences between samples did not interfere with the detection of fennel origin, which is consistent with many current studies based on FT-NIR spectral data to distinguish the geographical origin of plant foods^37,38^.

### Feature extraction

Principal Component Analysis (PCA) is a classic unsupervised feature extraction algorithm that can reduce the dimensionality of the data^39^. PCA compresses the highly correlated original variables into a few new variables through linear transformation, seeking new variables (that is, principal components) that can maximize the data structure characteristics of the original variables. The first variable has the largest variance and becomes the first principal component, followed by the second variable, and so on.

In this study, 221 samples were randomly selected from 316 samples as the training set and 95 samples as the test set in a ratio of 7:3 for each trial. The cross-validation method was used to randomly divide the data set into training and validation sets, and the randomly generated subsamples were repeatedly applied for training and validation. The PCA algorithm (MATLAB R2019b) was used to extract the features of Rubberband baseline correction spectral data.

### Classification model

**Figure 1:**
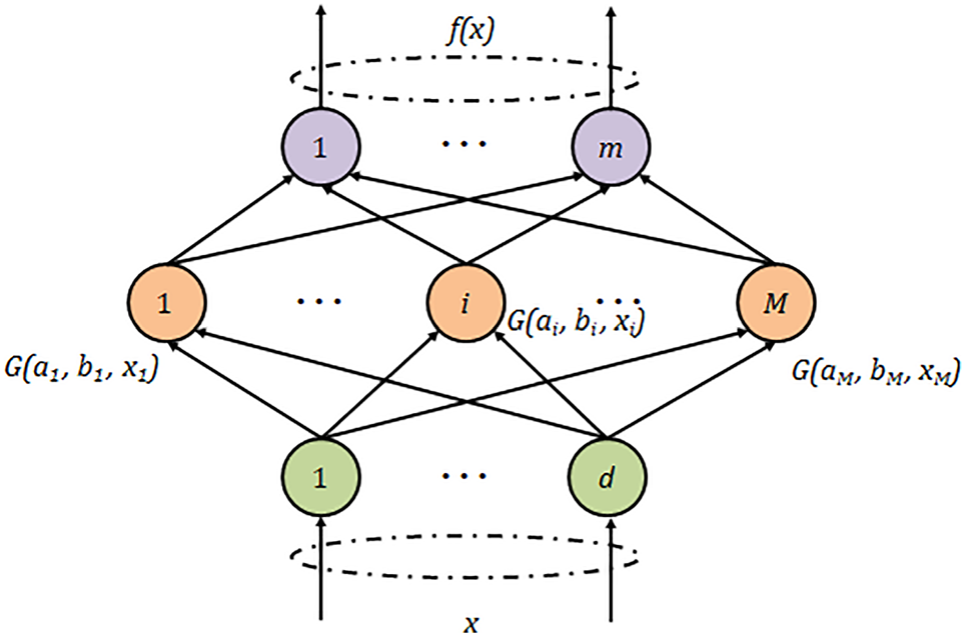
Neural network structure of ELM.

As a feedforward neural network, the ELM (Extreme Learning Machine) has shown good performance in the application of dataset classification in recent years^31^. The neural network structure of the ELM model is shown in Fig. 1, and the inputs and outputs of the ELM are x and f(x), respectively. The sampled sample data is $x_i$, and the activation function is G($a_j$,$b_j$,x), where $a_j$ and $b_j$ denote the connection weights and bias values between the input and hidden layers, respectively. Unlike traditional neural network learning algorithms, the ELM not only tends to reach the minimum training error but also the minimum output weight norm. Bartlett’s theory shows that for feedforward neural networks if the training error is minor and the weight norm is smaller, the network tends to have better generalization performance^40^. Significantly, ELM has a faster learning speed with great performance; thus, we choose ELM as the first kind of classification model, the activation function kinds are set to Sigmoid, Sine, Hardlim, and the number of neurons in the hidden layer is set to 100. Three models are obtained: ELM-sig, ELM-sin, ELM-hardlim.

The BPNN algorithm is one of the most widely used neural network models, which is a multi-layer feedforward network trained by error backpropagation. BPNN has good ability for self-learning, self-adaptation and generalization, and has excellent effects when used in binary classification^41^. In recent years, BPNN has been widely used in food research, biomedicine and other fields^42–44^. In this study, we choose BPNN as the second kind of classification model, set tansig and logsig as the activation function of the hidden layer, and the output layer function is purelin. The training function of the neural network is trainlm. Set the number of neural network training to 500 times, the learning rate parameter to 0.01, and the learning target parameter to 0.1. Two models are obtained: BPNN-tansig and BPNN-logsig.

The RNN introduces state variables to store past information, and uses state variables with the current input to determine the current output. LSTM is a typical variant of RNN, it is particularly suitable for the classification of sequential data. In recent years, LSTM has been widely used in biomedical classification task^45^. We choose RNN as the third classification model. We set the number of hidden layers to 3, the probability of the dropout layer to 0.5, the learning rate to 0.0001, the batch size to 8 and the epoch size to 20.

The transformer network is developed based on the attention mechanism, which consists of an encoder and decoder for handling long-term dependencies in sequence-to-sequence tasks. In addition, it has excelled in classification tasks^46^. Therefore, we choose it as the fourth classification model. We apply dropout to the output of each sublayer, which is then added to the input of the sublayer and normalized, where the dropout is set to 0.5. Set the learning rate to 0.00001, the batch size to 8 and the epoch size to 40.

The CNN has two features of sparse connectivity and weight sharing, so it is less computational than multilayer perceptron (MLP) and its performance is better. In the field of spectroscopy, the one-dimensional (1D) CNN models are used to learn and predict spectra with good performance^47^. In this study, it is used as the fifth classification model. We choose ReLU as the activation function and set the learning rate to 0.001, the probability of the dropout layer to 0.5, the batch size to 16 and the epoch size to 75.

GAN consists of two artificial neural networks, G and D. The two networks compete with each other to achieve improvement in model performance during training. GAN shows high accuracy in food detection and classification^28^. So we choose it as the sixth classification model. In the fennel classification experiments, we need to train four models from two origins, including G1, G2, D1 and D2. We use G to generate the false spectral data, and then use D to determine the generated data is the probability of being true, and the next step is to use the BCELoss loss function to measure the difference between the probability and the true label. We select Adam as the optimizer of G and D and set the training iteration period to 500 and the learning rate to 0.0001.

In summary, eleven models are obtained in this study: ELM-sig, ELM-sin, ELM-hardlim, BPNN-tansig, BPNN-logsig are compiled in MATLAB, and RNN, Transformer, CNN, GAN ELM, BPNN are compiled in Python.

## Results

### Spectral analysis

FT-NIR spectroscopy is generated by the transition of molecular vibration from the ground state to the higher energy level in overtones and combination modes due to the non-resonance of molecular vibration. It mainly records the overtones and combination modes absorption of the vibration of hydrogen-containing groups, covering the composition and molecular structure information of most types of organic compounds^48^. Due to the difference in the absorption wavelength of organic compounds, FT-NIR spectroscopy is suitable for the determination and analysis of hydrocarbon organic compounds.

**Figure 2:**
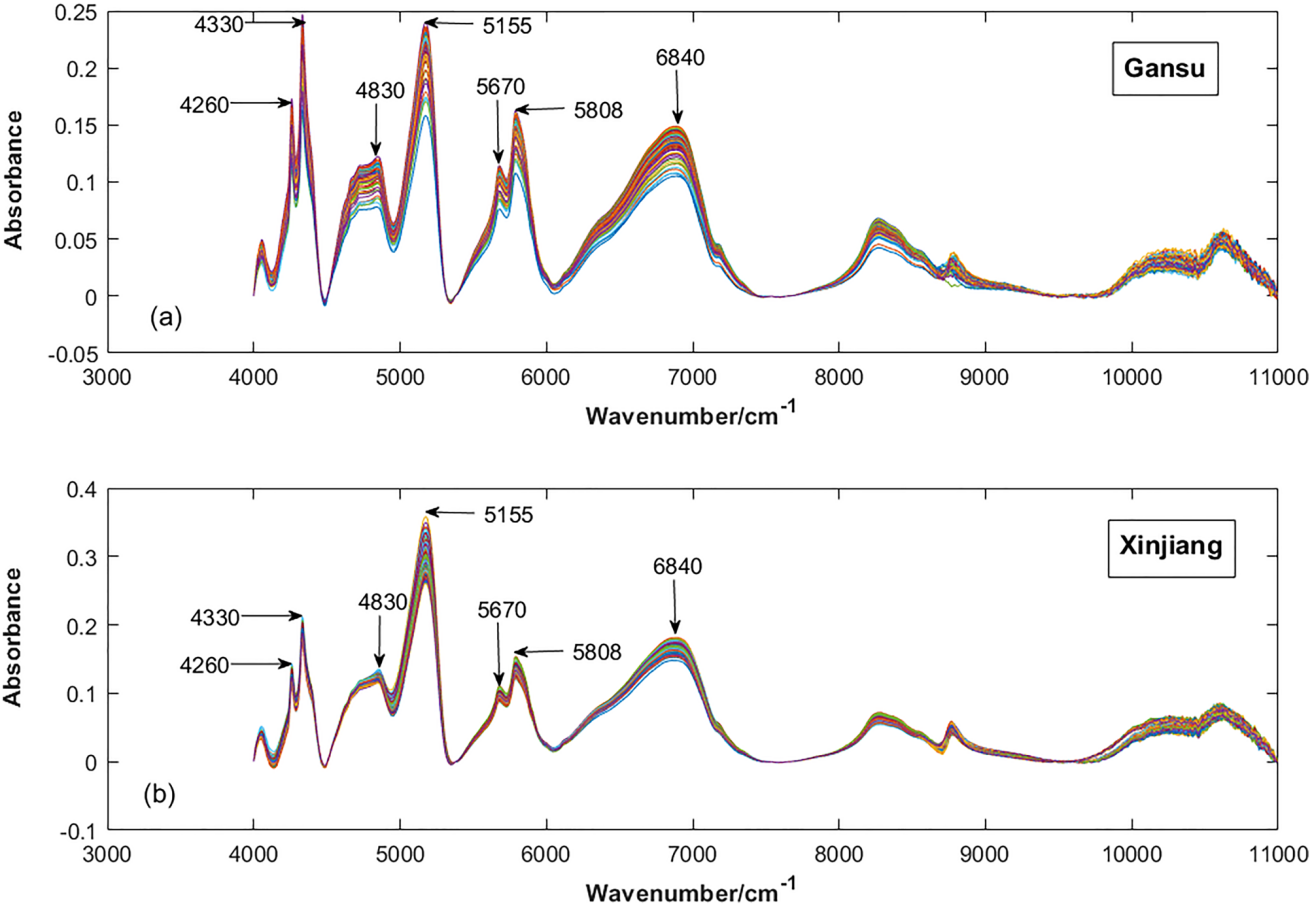
The NIR spectrum of the fennel.

Figure 2 is the FT-NIR spectrum of the fennel after baseline correction. (a) is the sample of fennel from Yumen, Gansu, and (b) is the sample of fennel from Turpan, Xinjiang. In Fig. 2, it can be observed that there are peaks at 4260, 4330, 5155, 5670, 5808, 6840 $\mathrm {cm}^{-1}$, etc., and the peak shape is relatively sharp, indicating that the information contained in this place is relatively rich. The region between 5000 and 4000 $\mathrm {cm}^{-1}$ is mainly the frequency absorption of C–H, and this region is generally considered to be the characteristic absorption band of sugar, protein and starch^49–51^. In the vicinity of 5155 $\mathrm {cm}^{-1}$, there are two vibrations due to O–H stretching and O–H deformation, which can be speculated as water absorption zone^52^, which may be because the dried fennel still contains a small amount of water. The FT-NIR spectrum at 7500 and 5500 $\mathrm {cm}^{-1}$ is mainly the stretching vibration of C–H in $\mathrm {CH}_{3}$– and $\mathrm {CH}_{2}$– groups. It can be seen from Figure 1 that the peak distribution and trend of the FT-NIR spectra of the samples of Yumen fennel in Gansu and Turpan fennel in Xinjiang are similar, indicating that the sample composition and related information are similar. Spectral peaks of individual bands (such as 4330 and 5155 $\mathrm {cm}^{-1}$, etc.) are different, indicating that there are significant differences in the percentage content of various oils and substances such as water content contained in fennel from different origins, which is also consistent with the conclusions reached in some previous studies^53,16^. Therefore, it is feasible to use FT-NIR spectroscopy to classify and identify fennel in Gansu and Xinjiang. As can be seen from Table 1, the main substances in fennel include nutrients such as proteins and fatty acids, polyphenols (hydroxyl and –CH groups are mostly phenols) other antioxidant substances and flavonoids, etc.^12,15,54^.

**Table 1:** Waveband and composition distribution of major NIR peaks of the fennel.

Wavenumber ($\mathrm {cm}^{-1}$)	Assignment	Substances related including in fennel
4260	O–H, N–H and C–O bands	Essential oils
4330	C–H of Lipids	Hydroxy fatty acids
4830	Amide	Proteins
5155	C–H, N–H, O–H of water molecule	Water
5670	–${\mathrm{CH}_{2}}$	Steroids
5808	–$\mathrm {CH}_{3}$ of polyphenols	Polyphenols
6840	polyamides	Flavonoids

### PCA feature extraction

**Figure 3:**
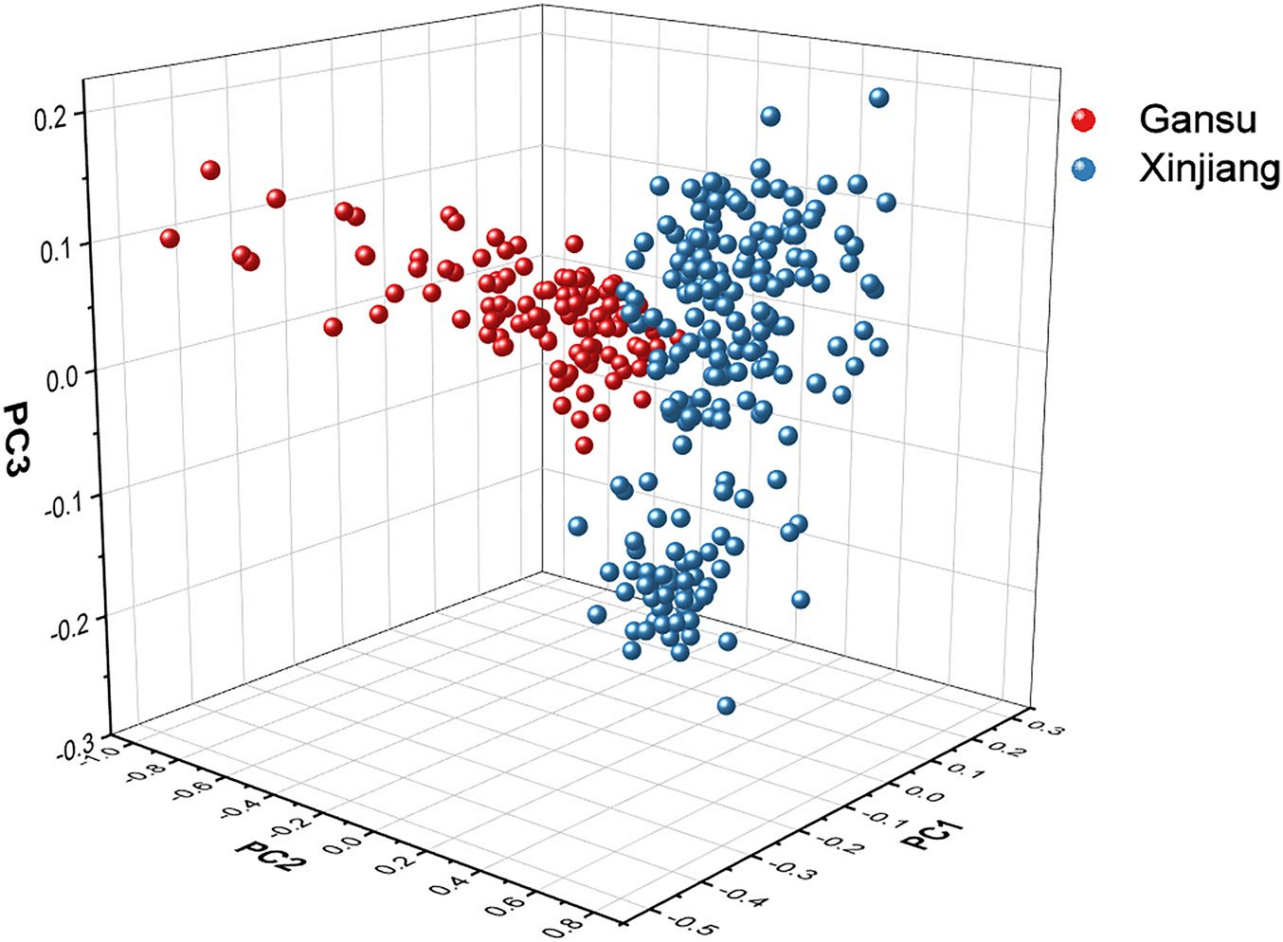
Three-dimensional scatter plot of three principal components of the fennel FT-NIR spectroscopy.

If the number of principal components is too large, it is easy to introduce noise and redundant data^55^. In this experiment, we selected the first 6-dimensional data of the fennel spectral data as the principal components, and the cumulative variance contribution rate has reached 99.26%. The specific information of the contribution rate is shown in Table 2. Their first three PCs score are shown in Fig. 3, and the drawing software used is Origin Pro 2019b.

**Table 2:** The contribution rates of the first six features in PCA.

Principal component	Contribution rate(%)	Cumulative contribution rate(%)
1	85.93	85.93
2	6.22	92.14
3	4.80	96.95
4	1.45	98.40
5	0.51	98.91
6	0.35	99.26

### Model evaluation

In this study, the Gansu fennel sample is regarded as a positive sample, and the Xinjiang fennel sample is regarded as a negative sample. After eleven models of ELM-sig, ELM-sin, ELM-hardlim, BPNN-tansig, and BPNN-logsig, RNN, Transformer, GAN, BPNN, CNN, ELM are obtained, each model running 30 times, record the specificity, sensitivity, accuracy, and model of every run time, 30 times experiment after calculating the average of the index record in Table 3.

**Table 3:** Experimental performance of each model comparison results.

Languages	Model	Sensitivity (%)	Specificity (%)	Accuracy (%)	Run time(s)	AUC
MATLAB	ELM-sig	97.89	99.18	98.73	0.05	0.98
	ELM-sin	97.63	99.51	98.84	0.04	0.98
	**ELM-hardlim**	**100.00**	**100.00**	**100.00**	**0.05**	**1.00**
	BPNN- tansig	95.92	97.47	96.91	0.51	0.96
	BPNN- logsig	98.07	97.17	97.43	0.44	0.97
Python	RNN	100.00	100.00	100.00	2.01	1.00
	Transformer	100.00	100.00	100.00	8.47	1.00
	GAN	100.00	100.00	100.00	126.22	1.00
	BPNN	98.75	93.33	97.89	0.45	0.96
	CNN	62.96	65.15	64.51	2.29	0.64
	**ELM**	**100.00**	**100.00**	**100.00**	**0.03**	**1.00**

Significant values are in [bold].

It can be seen from Table 3 that ELM- and BPNN-based algorithms have similar results for evaluation indexes in different programming languages, and even the running time of Python is slightly faster than that of MATLAB. The accuracy reflects the proportion of the number of correctly classified samples to the total number of samples. The accuracy of the BPNN model is more than 96%, and the accuracy of the ELM, RNN, GAN and Transformer models are above 98%, especially the ELM model with Hardlim as the activation function has the best classification effect with 100% accuracy and the fastest running time. In the ELM model, the selected activation function is different, and the running time is also different, but the difference is not obvious. Among them, the ELM model with Sine as the activation function has the shortest running time, indicating that the model has the fastest classification speed. It can be clearly concluded from the results in Table 3 that the running time of the ELM model is shorter than that of the BPNN and CNN models, and the classification accuracy of the ELM model is higher. In addition, the ELM model is faster in achieving the same high classification accuracy as RNN, GAN and Transformer models.

**Figure 4:**
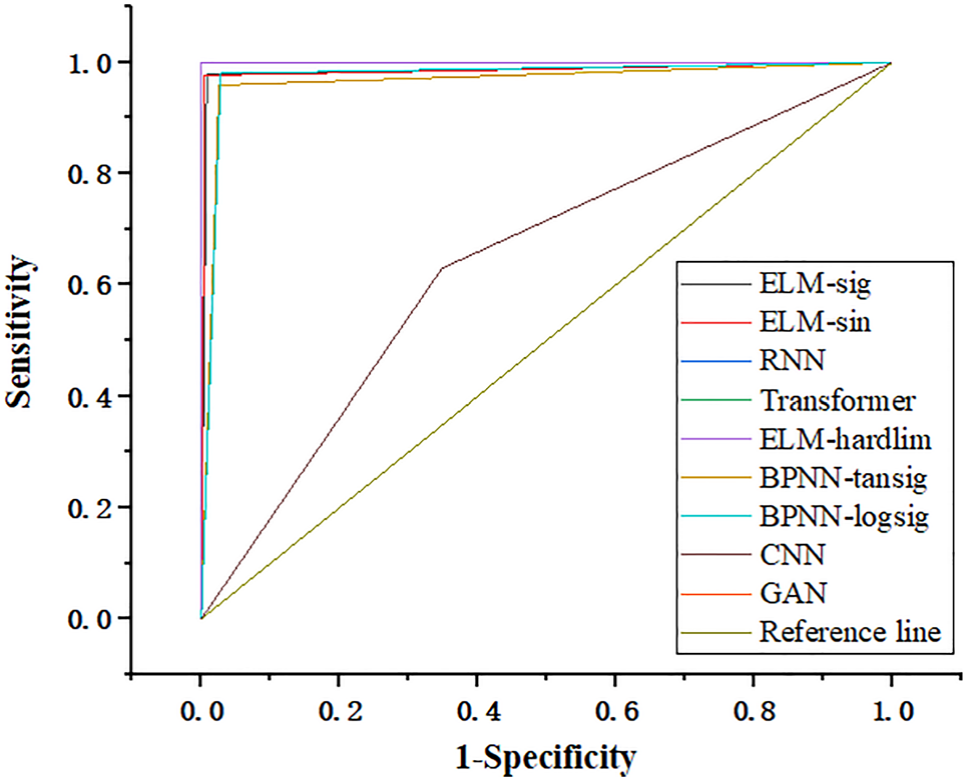
AUC curves of each model.

In order to further verify the reliability of the model’s classification of fennel origin, we introduced a receiver operating characteristic curve (ROC) curve to evaluate it. The horizontal and vertical coordinates of the ROC curve represent the specificity and sensitivity of the model, and the area under the curve (AUC) can be used as an index to evaluate the classification effect of the model. Where specificity indicates the proportion of correctly classified unqualified samples to the number of unqualified samples, and sensitivity is the proportion of correctly classified qualified samples to the number of qualified samples. The closer the AUC value is to 1, the better the effect of the classifier^56^. We used OriginPro 2019b to draw the ROC curve of each model in Fig. 4, and further obtained the average AUC value of each model. The results are recorded in Table 3. It can be seen that the AUC values of the ELM, RNN, Transformer, GAN and BPNN models are greater than 0.96, which indicates their high reliability in classifying fennel origins in Yumen, Gansu and Turpan, Xinjiang. In Fig. 4, the AUC values of the ELM model are all greater than those of the BPNN model and CNN models, and the AUC value of the RNN, Transformer, GAN and ELM models with Hardlim as the activation function all reach 1.00, but the classification speed of the ELM model is the fastest, only 0.05s. In summary, the ELM model runs fast, has a high accuracy rate, a large AUC value, and the classification effect is better than the BPNN, RNN, Transformer, GAN and CNN models.

## Conclusion

The kinds and contents of antioxidant substances, phenols, flavonoids and other substances in the fennel from the different producing areas are different^19^. In this study, the FT-NIR spectroscopy of the fennel combined with the machine learning algorithm is used for the first time to classify the fennel from different producing areas. The results show that the classification accuracy of the BPNN model is above 96%, the classification accuracy of the ELM, RNN, GAN and Transformer models is above 98%. The classification accuracy of the ELM model with the hardlim as the activation function can reach 100%. The classification speed of the ELM model is significantly faster than that of the RNN, GAN and Transformer models, with an average classification speed of 0.05s after 30 experiments. The experiments show that our proposed ELM model is more lightweight and faster in detection speed with guaranteed detection accuracy. Compared with the current mainstream deep learning models, the ELM model can combine the advantages of both high performance and low time cost, and at the same time solves the problem of poor detection accuracy and large time cost spent in the detection of massive data, which is more valuable for large-scale classification tasks in practical applications. The results of this study show that the use of the FT-NIR spectroscopy with simple sample preparation and fast detection speed, combined with machine learning algorithms, can achieve rapid identification of the fennel from different origins, thereby helping consumers better identify high-quality products and preventing unscrupulous merchants from shoddy behavior helps the market supervise related industries. In addition, this research technology should be introduced to other food classifications of different origins, providing new ideas for the intelligent supervision of the origin of various foods in the future.

## Supplementary Information

Supplementary Information.

## Data Availability

All data generated or analysed during this study are included in this published article [and its supplementary information]. S1 File. In this file, 200 samples were collected from Lianyungang Kaihao Tong Trading Co. in Xinjiang, and 116 samples were collected from Gansu Yumen Xiaosannong Taobao online store.
